# Editorial Perspective: COVID‐19‐related publications on young people’s mental health – what have been the key trends so far and what should come next?

**DOI:** 10.1111/jcpp.13615

**Published:** 2022-04-19

**Authors:** Samuele Cortese, Michel Sabe, Marco Solmi

**Affiliations:** ^1^ 7423 Centre for Innovation in Mental Health School of Psychology Faculty of Environmental and Life Sciences University of Southampton Southampton UK; ^2^ 7423 Clinical and Experimental Sciences (CNS and Psychiatry) Faculty of Medicine University of Southampton Southampton UK; ^3^ Solent NHS Trust Southampton UK; ^4^ Hassenfeld Children's Hospital at NYU Langone New York University Child Study Center New York City NY USA; ^5^ Division of Psychiatry and Applied Psychology School of Medicine University of Nottingham Nottingham UK; ^6^ Division of Adult Psychiatry Department of Psychiatry University Hospitals of Geneva Thonex Switzerland; ^7^ 6363 Department of Psychiatry University of Ottawa Ottawa ON Canada; ^8^ Department of Mental Health The Ottawa Hospital Ottawa ON Canada; ^9^ 6363 Ottawa Hospital Research Institute (OHRI) Clinical Epidemiology Program University of Ottawa Ottawa ON Canada

## Abstract

In this Editorial Perspective, we take a systematic look at the overall nature of the Covid‐19 related research on mental health in children and young people, to gain insight into the major trends in this area of research and inform future lines of investigation, clinical practices, and policies. By means of state‐of‐the‐art scientometric approaches, we identified 3,692 relevant research outputs, mainly clustering around the following themes: (a) mental health consequences of the Covid‐19 pandemic in children and young people; (b) impact of the pandemic on pre‐existing psychiatric disorders; (c) family outcomes (i.e., family violence and parental mental health); and (d) link between physical and mental conditions. Only 23% of the retrieved publications reported new data, the remaining ones being reviews, editorials, opinion papers, and other nonempirical reports. The majority of the empirical studies used a cross‐sectional design. We suggest that future research efforts should prioritise: (a) longitudinal follow‐up of existing cohorts; (b) quasi‐experimental studies to gain insight into causal mechanisms underlying pandemic‐related psychopathology in children and young people; (c) pragmatic randomised controlled trials (RCTs) to test evidence‐based intervention strategies; and (d) evidence‐based guidelines for clinicians and policymakers.

Now that we have entered the third year of the pandemic, it seems appropriate to stand back and take a systematic look at the overall nature of the Covid‐19 related research that we, as a scientific community of researchers and clinicians in the field, have produced over the past two years, to inform future lines of investigation, clinical practices, and policies. To this end, we quantitatively and critically analysed the literature focused on mental health in children and young people related to the Covid‐19 pandemic. We used scientometric approaches and dedicated software outputs (Mingers & Leydesdorff, 2015) to conduct a *systematic mapping* of 3,692 research identified during a systematic search of the literature, by examining the frequency with which two documents are cited together by other documents (i.e., co‐citation index; for the protocol seehttps://osf.io/cjntd/?view_only=3363c636d9244a88899ce50abcb07ac3). First, we looked at the content of the papers. Our systematic knowledge map of publications (Figure 1) allowed us to extract four major trends in pandemic‐related child and young person mental health literature. The first major trend focused on the mental health consequences of the Covid‐19 pandemic, represented by a number of clusters of co‐cited papers in Figure 1. One cluster focused *on child and adolescent mental health professionals*, a second on *preschool children*, and a third on *college/university students*. Other clusters included the impact of the pandemic on *physical activity*, *suicide risk*, and *prenatal and perinatal factors*. The second major trend related to the impact of the pandemic on pre‐existing psychiatric disorders, with clusters on *autism spectrum disorder*, *substance use disorder*, and *eating disorder*. The third trend pertains to family outcomes, with clusters focused on *family violence* and *parental mental health*. The final trend focused on somatic consequences with a cluster on *multisystem inflammatory syndrome*.

**Figure 1:**
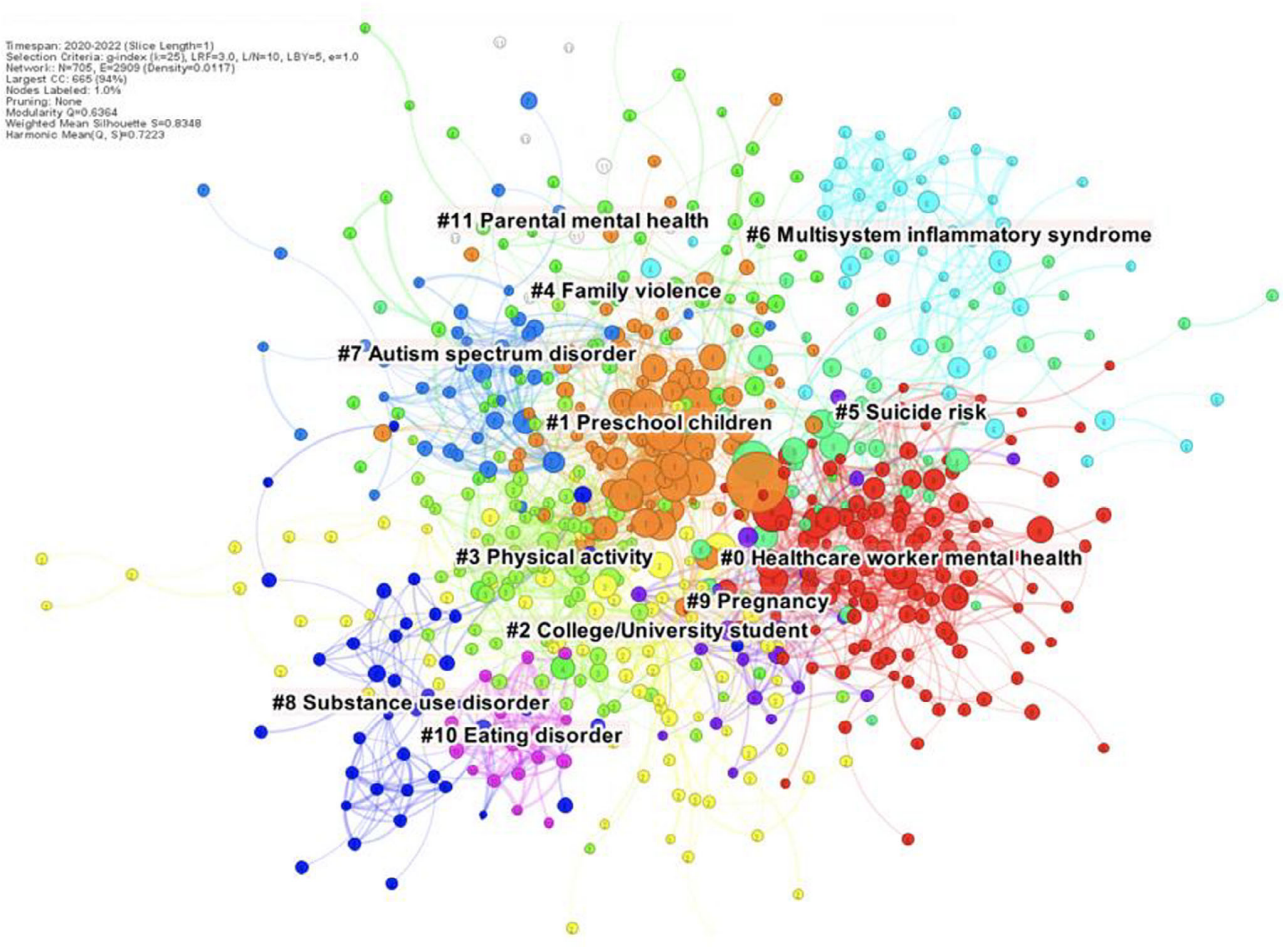
References co‐citation network

An analysis of the type of publications was very revealing. Of those the nature of which could be definitively ascertained, only 851 papers provided new data. This included 530 cross‐sectional, 221 longitudinal studies and 93 trials, encompassing 12 randomised controlled trials (RCTs) of mental health interventions relevant to Covid‐19. The remainder included 439 reviews (including 104 systematic reviews and 57 meta‐analyses), 176 case reports or case series, and 153 editorials.

What does this tell us about (a) research on mental health of children/young people during the pandemic already conducted, and (b) where future resources should be targeted? In terms of *contents/topics*, many papers have focused on topics of importance based on clinical anecdote: the increase in adolescent eating disorders (Solmi, Downs, & Nicholls, 2021) and suicide risk (Asarnow & Chung, 2021), family violence and maltreatment (Sonuga‐Barke & Fearon, 2021), and the impact of isolation on autism spectrum disorder (Alonso‐Esteban, López‐Ramón, Moreno‐Campos, Navarro‐Pardo, & Alcantud‐Marín, 2021). Some disorders such as sleep disorders (Sharma, Aggarwal, Madaan, Saini, & Bhutani, 2021) and depression or anxiety (Racine et al., 2021) are not represented as clusters – probably because the number of citations of relevant papers has not yet reached a sufficient level. There is a welcome focus on the impact of the pandemic on the social networks around the child (family, and health care including mental health care, professionals). Interestingly, we also found a cluster on pregnancy and the transgenerational risk of mental disorders after the current pandemic will need to be monitored (Arango et al., 2021). We found an additional intriguing cluster on Covid‐19 and multisystem inflammatory systems. We hope that this will stimulate additional lines of research on the link between mind and body (Cortese, Arrondo, Correll, & Solmi, 2021) aimed at understanding to which extent the virus SARS‐CoV‐2 can contribute to (neuro)psychiatric dysfunctions in children and young people via possible direct effects on the brain (Swanson & Volkow, 2021) and indirectly via the psychological impact of alterations in the body.

In terms of types of papers, it is clear that primary empirical research studies represent a minority of papers published so far. This is probably not surprising, considering the speed with which the pandemic hit, the impact of the pandemic on many research programmes around the globe, and the strains placed on the personal lives of researchers. In this context, efforts of investigators who conducted primary empirical research are to be commended. Certainly, opinion pieces, editorials, and narrative reviews of the literature have played an important role in the initial phases of the pandemic, when empirical data were scant, and will of course keep on playing an important role in critically appraising available studies. However, we hope to see a more balanced ratio between primary empirical research and opinion articles in the upcoming phases of the pandemic. As most of the available empirical studies in the field are cross‐sectional, with the methodological limitations of this design, two types of studies seem highly relevant in terms of public health. First, it will be important to continue the longitudinal follow‐up of existing cohorts, to continuously assess the impact of the pandemic on the lives of children and their families during and beyond the pandemic. In this regard, the Co‐Space study following school‐aged children and young people aged 4–16 years (at the beginning of the study) on a monthly basis is one (certainly not the only) excellent example. The last report provided an overview of monthly data from 9,161 parents/carers (Raw et al., 2021). The field will clearly benefit from continuous longitudinal data, particularly when collected across many countries, to gain insights into the possible impact of different measures and approaches used to contain the spread of the virus, as well as the risk and protective factors for mental health in children and adolescents across different geographic settings (Solmi et al., 2022). Second, we hope to see an expansion in the small pool of studies evaluating interventions, once again including a multinational perspective and possibly adopting pragmatic designs (pragmatic RCTs). On the one hand, RCTs will continue to provide evidence on the efficacy and tolerability/safety of interventions to tackle the burden of mental health conditions in children, young people, and their families. On the other hand, they will also provide compelling evidence shedding light on the causal mechanisms initially suggested in cross‐sectional studies. Third, the additional role of natural experiments in providing insights into causal processes has been elegantly highlighted in this *Journal,* pointing out that ‘*A careful selection of regions differing in lockdown type, intensity and timings will allow a powerful combination of a between‐group comparison of regional variations in mental health rates and a within‐group analysis of the temporal covariation between fluctuating levels of mental health problems and changes in lockdown policy, providing a strong test of the causal role of lockdown in determining mental health’* (Sonuga‐Barke & Fearon, 2021). In addition to risk factors, resilience factors (e.g., the role of physical activity) should also be explored in more depth. Fourth, many of the guidance documents published about care during the pandemic were based mainly on expert consensus (Cortese et al., 2020). Continuously accumulating empirical evidence should inform future guidance/guidelines. An important additional step will be provided by research inspired by the implementation science approach, promoting the integration of empirical findings and evidence into healthcare policy and practice.

In summary, while there have been a large number of publications on Covid‐19 and mental health in children and young people, there is a pressing need to conduct additional types of studies. We hope that the insights provided by this systematic mapping analysis will contribute to inform funding bodies on research priorities in the field, with the ultimate goal to provide support to children and their families during this and possibly future pandemics, based on the best available science.
